# Mechanisms of Salt and Drought Stress Responses in Foxtail Millet

**DOI:** 10.3390/plants14081215

**Published:** 2025-04-15

**Authors:** Gemechu Nedi Terfa, Wenqiu Pan, Longjiao Hu, Junwei Hao, Qinlong Zhao, Yanzhe Jia, Xiaojun Nie

**Affiliations:** 1 State Key Laboratory of Crop Stress Resistance and High-Efficiency Production, College of Agronomy and Yangling Branch of the China Wheat Improvement Center, Northwest A & F University, Yangling 712100, China; gemechu.nedi@ambou.edu.et (G.N.T.);; 2 Department of Plant Science, School of Agricultural Science, Ambo University, P.O. Box 19 Ambo, Ethiopia

**Keywords:** drought stress, foxtail millet, gene function, molecular mechanisms, salt stress

## Abstract

Salt and drought are destructive abiotic stresses that severely impact crop production and productivity, posing an increasing threat to global food security, particularly as their occurrence rises annually due to climate change. These salt and drought stresses adversely affect plant growth and development, leading to significant reductions in crop yields. Foxtail millet (*Setaria italica*) exhibits various adaptive mechanisms, including enhanced antioxidative systems, osmotic adjustment through osmolyte accumulation, and root system modification, which facilitate its tolerance to stressors. These traits underscore its significant potential for breeding climate-resilient crops to address food security and climate change challenges. Understanding the molecular basis of salt and drought tolerance mechanisms is essential for breeding or genetically engineering foxtail millet varieties with enhanced salt and drought tolerance, as well as improved yield potential. This review systematically overviewed the research progress and current status of the mechanisms underlying foxtail millet’s tolerance to salt and drought stress from the perspectives of physiological, biochemical, and molecular responses. Additionally, it provides some future perspectives that will contribute to further deciphering the genetic mechanisms governing salt and drought tolerance, as well as further genetic improvement in foxtail millet.

## 1. Introduction

The agricultural sector is currently facing a significant challenge due to climate change, which is expected to reduce agricultural output. Consequently, increasing global food insecurity and rising food prices are anticipated outcomes of reduced productivity [1,2]. Key manifestations of climate change, such as salinity, heat, drought, and cold stress, are primary contributors to abiotic stress. Among these factors, salinity and drought are particularly critical, as they reduce the extent of usable arable land and significantly impair crop productivity, particularly in semi-arid and arid regions [3]. These issues have had a detrimental impact on global food security [4,5]. Similarly, elevated temperatures adversely affect crop yields [6,7]. To address these challenges, researchers are actively investigating the genetic and genomic attributes of crops with enhanced tolerance to these stressors [8]. Certain orphan crops have been identified as climate resilient, with foxtail millet emerging as a notable example due to its adaptability to changing climatic conditions [9].

Foxtail millet (*Setaria italica*), a relatively underutilized crop, has the potential to alleviate food and nutritional insecurity in the context of a growing global population. Nutritionally, it surpasses staple cereals such as wheat and rice in multiple aspects [10]. Although it occupies a minor share of global food grain production, its importance in enhancing food and nutritional security deserves greater recognition. Its value lies in its ability to thrive on marginal agricultural lands that would otherwise remain unproductive. Despite its limited attention, foxtail millet has historically served as a staple food in many semi-arid and arid tropical regions where the cultivation of major crops is constrained by low rainfall and poor soil quality [11,12].

Foxtail millet ranks second in global millet production, following pearl millet. However, its productivity is significantly higher, with yields of 3.9 and 5.8 tons per hectare under rainfed and irrigated conditions, respectively, compared to pearl millet’s yields of 2.2 and 2.8 tons per hectare under similar conditions [13]. Foxtail millet requires only about one-quarter of the water needed for staple crops such as rice [14,15] and demonstrates significantly higher water use efficiency compared to maize and wheat. For instance, producing 1 g of above-ground dry biomass in foxtail millet requires only 257 g of water, whereas 470 and 510 g were required by maize and wheat, respectively [16].

Foxtail millet is a resilient cereal crop that is well adapted to challenging environments, demonstrating superior tolerance to abiotic stresses compared to other cereals. Under stressed conditions, the plant activates interconnected regulatory pathways that facilitate adaptation to environmental challenges. Abiotic stress significantly affects plant physiology and cellular processes, often disrupting them. While some changes are non-adaptive, many are adaptive and enhance the plant’s resistance to abiotic stress. Recent research has extensively focused on uncovering the physiological and molecular factors that contribute to crop tolerance to abiotic stresses, including foxtail millet [17,18]. The abiotic stress responses in foxtail millet involve various morphological, physiological, biochemical, and molecular mechanisms, enabling the plant to cope with challenges such as salinity, drought, heat and other stresses [19,20]. Furthermore, foxtail millet adapts to abiotic stresses by increasing biochemical activities, including elevated antioxidant levels, reactive oxygen species (ROS) regulation, and the activation of ROS-scavenging enzymes. Additionally, it enhances the activities of catalase and superoxide-related enzymes while promoting the synthesis of osmolytes and stress-associated proteins [21,22]

While foxtail millet exhibits superior drought tolerance compared to other cereal crops, it can still experience substantial yield losses under drought stress due to disruptions in physiological processes such as photosynthesis and nutrient uptake [23]. The extent of yield loss varied by cultivar. For instance, the variety Qitoubai experienced a 31% yield loss under drought conditions, while the variety Jingu21 showed a 54% reduction [24]. Similarly, soil salinity can reduce foxtail millet yields by 11–85% [15]. In one study, the salt-tolerant variety BD-881 exhibited a 12% yield reduction, while the salt-susceptible variety BARI Kaon-1 had a 38% reduction [25]. Furthermore, germination rates among foxtail millet genotypes under high salt stress vary widely, ranging from 0% to 90% [26]. These findings underscore the substantial genetic variation in foxtail millet genotypes in response to salt and drought stresses.

Although considerable efforts have been made to identify genes and traits associated with salt and drought tolerance in foxtail millet, the underlying mechanisms remain insufficiently explored. So far, several salt- and drought-responsive genes have been functionally characterized in foxtail millet, contributing to enhanced salt and drought stress resistance during critical growth stages [27,28]. Understanding these stress responses is essential for addressing challenges related to climate change, food security, and sustainable agriculture. This knowledge helps develop better breeding strategies, improves crop resilience, and enables cultivation in marginal environments, ultimately fostering a more sustainable agricultural system [22,29].

This review aims to summarize the adaptive mechanisms of foxtail millet in response to salt and drought stresses, including stress-signaling pathways, gene regulation, and antioxidant systems. The primary objective is to identify genes associated with salt and drought tolerance, which may be utilized in future genetic modification or breeding programs to enhance stress tolerance in foxtail millet and other crops.

## 2. Response to Salt and Drought Stresses

**Figure 1:**
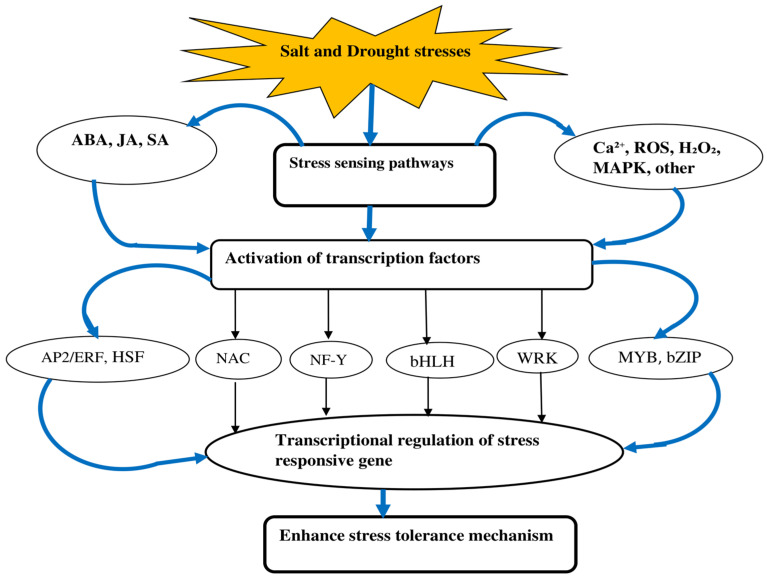
Schematic representation of stress-responsive genes and stress tolerance mechanisms of foxtail millet under salt and drought stress.

Under salt and drought stresses, plants undergo various morphological, biochemical, physiological, and molecular changes, which can lead to cellular damage and impaired growth [5,30]. Both salt and drought stress negatively affect foxtail millet at both the biochemical and morphological levels, potentially reducing its yield. Studies indicate that membrane lipid peroxidation is significantly impacted under salt and drought stresses [31], resulting in cell membrane damage and disintegration. This damage is primarily caused by the accumulation of reactive oxygen species (ROS) [5], including hydrogen peroxide (H_2_O_2_), superoxide anions (O_2_^−^), hydroxyl radicals (•OH), alkoxyl radicals, and nitric oxide [32,33]. At the morphological, physical, biochemical, and molecular levels, foxtail millet uses a variety of techniques to regulate the effects of salt and drought [18] (Figure 1). The mechanisms utilized by the plant to respond to salt and drought are largely similar.

### 2.1. Transcription Factor Response

Foxtail millet exposed to salt and drought stress environment conditions detects stress signals through specialized receptors and engages in complex signal transduction pathways. This process leads to the activation of stress-inducible transcription factors [18]. A transcription factor is a protein that binds to DNA and controls gene expression by activating or inhibiting transcriptions [34]. Its role is to regulate when and where genes are expressed, ensuring they are turned on or off at the right time and in the appropriate amount throughout the cell’s life and the organism’s development [35]. Genomic analyses and transgenic approaches in foxtail millet have been employed to identify and characterize transcription factors contributing to drought and salt stress responses, resulting in the establishment of relevant datasets [19,36,37]. In foxtail millet, when the plant faces salt and drought stress, it significantly increases the activity of certain transcription factors from groups such as Dehydration Responsive Element-Binding protein (DREB), Myeloblastosis (MYBs), Basic Helix–Loop–Helix (bHLH), Basic Leucine Zipper (bZIP), NAC, Nuclear Factor Y (NF-Y), heat shock factor (HSF), WRKY, and APETALA2/Ethylene Responsive Factor (AP2/ERF) [19,36,37,38]. Researchers have identified several transcription factors that contributed to both drought and salt stress tolerance (Figure 1).

To investigate the role of AP2/ERF transcription factors in foxtail millet, a comprehensive genome-wide analysis was conducted using silicon methods [39]. The study revealed that the SiAP2/ERF-069, SiAP2/ERF-103, and SiAP2/ERF-120 genes have the potential to develop drought- and salt stress-resistant crops in an ABA-dependent manner. Similarly, NAC, like transcription factors, was found to be induced under drought and salt stress conditions in foxtail millet [40,41]. Furthermore, overexpressing SiNAC110 in transgenic *Arabidopsis* improved the plant’s tolerance to both drought and salt stresses. SiNAC110 likely enhances stress tolerance by regulating the expression of genes associated with proline biosynthesis, ion homeostasis, and osmotic balance maintenance, all through an ABA-independent signaling pathway [40]. In other studies, DREB-type transcription factors control the expression of genes that are activated by salt and drought stress by binding to the DRE/CRT cis-elements in the promoter regions. Consequently, the expression of SiARDP in *Arabidopsis thaliana* improved salt and drought tolerance during seed germination and seedling development [42]. Another study confirmed that the SiARDP protein can bind to SiASR4, leading to increased SiASR4 gene expression in plants overexpressing SiARDP. Transgenic *Arabidopsis* and foxtail millet overexpressing SiASR4 exhibited enhanced tolerance to salt and drought stress. Additionally, the transcription of stress-responsive genes and those associated with reactive oxygen species scavenging was activated in SiASR4 transgenic plants. These findings suggest that SiASR4 plays a crucial role in adaptation to salt and drought stress, with its regulation mediated by SiARDP through an ABA-dependent pathway [43].

In foxtail millet, the nuclear factor-Y (NF-Y) transcriptional factors play a significant role in mediating responses to salt and drought stresses. A detailed study identified 39 NF-Y genes in foxtail millet, which are categorized into three subfamilies: SiNF-YA, SiNF-YB, and SiNF-YC [44]. Researchers found that overexpressing SiNF-YA1 and SiNF-YB8 in tobacco plants enhanced their stress tolerance. The overexpression of SiNF-YA1 improved drought and salt tolerance by activating stress-related genes, including NtERD10 and NtCAT1. Additionally, transgenic lines maintained a more stable relative water content, along with chlorophyll levels and the activity of key antioxidant enzymes such as superoxide dismutase, peroxidase, and catalase. Furthermore, the content of malondialdehyde remains relatively stable, contributing to enhanced drought and salt stress resistance [44,45,46].

### 2.2. Osmotic Adjustment

Osmotic adjustment is essential for maintaining cell turgor, which supports plant metabolic activity and sustains growth and productivity [47]. At the physiological level, the regulation of osmotic balance under abiotic stress is driven by the function of osmolytes [48]. Their subcellular compartmentalization facilitates the plant’s response to salt and drought stress by reducing the osmotic potential of water, thereby playing a crucial role in osmoregulation [49,50]. Osmolytes help to mitigate the adverse effects of abiotic stresses, either by directly preserving membrane integrity or indirectly by scavenging reactive oxygen species generated under stress [51]. The accumulation of cytoplasmic osmolytes reportedly reduces cellular water potential relative to the external water potential while alleviating high ionic strength in plants, including foxtail millet [52]. This mechanism promotes water influx and enhances water retention, which is vital for maintaining turgor pressure and supporting growth [53,54]. Both organic and inorganic ions are critical for osmotic tolerance, with higher plants frequently synthesizing organic solutes such as sugars and their derivatives [55]. Compatible osmolytes, including proline, soluble proteins, soluble sugar, polyols, and glycine betaine, are synthesized in response to osmotic stress, thereby playing significant roles in osmotic adjustment and the preservation of cellular integrity [56,57]. Furthermore, these compounds protect against membrane damage and enzyme inactivation in low-water environments, thus supporting normal cellular functions during salt and drought stress [58]. Proline accumulation is particularly critical for osmoregulation under salt and drought stress, as it helps cells retain water [47,59].

In foxtail millet, the overproduction of proline is essential for cellular homeostasis, water uptake, regulating osmotic pressure, and balancing redox reactions [60]. These processes are critical for restoring cellular structures and mitigating oxidative damage [22,61]. Wang et al. [62] identified a novel late embryogenesis abundant (LEA) gene, SiLEA14, in foxtail millet, which is activated under salt and drought stress. The study exhibited that transgenic lines of both *Arabidopsis* and foxtail millet with SiLEA14 exhibited significantly higher levels of free proline and soluble sugar compared to wild-type plants. As a result, the SiLEA14 transgenic foxtail millet exhibited enhanced osmotic stress tolerance. Similarly, transgenic *Arabidopsis* lines overexpressing SiNRX1 showed increased accumulation of proline. Moreover, these studies revealed that SiNRX1 overexpression improved *Arabidopsis* tolerance to salt and drought, resulting in a higher survival rate and enhanced growth performance [63]. Furthermore, foxtail millet exhibits notable phenotypic variations, including increased accumulation of compatible solutes such as proline under abiotic stress. This adaptation enhances its tolerance to drought and salinity, distinguishing it from other cereal crops, particularly those that are less adaptable to salt or drought stresses [64]. In another study, the genes for pyruvate orthophosphate dikinase (PPDK) and malate dehydrogenase (MDH) were identified and compared to those in other cereal crops such as rice, maize, sorghum, and brachypodium [65]. Foxtail millet contains a higher number of these genes, which are essential for osmotic adjustment and enable plants to withstand both salt and drought stress [66,67].

### 2.3. Oxidative Response

Oxidative stress is a condition that occurs when there is an imbalance between the production of reactive oxygen species (ROS) and the ability of an organism’s antioxidant system to detoxify them [68]. To mitigate ROS toxicity and safeguard against oxidative stress-induced damage, foxtail millet, like other plants, has evolved effective defense mechanisms [69]. Various protective enzymes, including superoxide dismutase (SOD), ascorbate peroxidase (APX), catalase (CAT), glutathione peroxidase (GPX), and peroxide reduction (PRX), play a crucial role in maintaining ROS homeostasis. These enzymes help neutralize excess ROS generated under salt and drought stress conditions, thereby preventing cellular damage and ensuring plant survival [70]. Foxtail millet demonstrates a strong oxidative stress response to salt and drought stress by enhancing its antioxidant defense mechanisms, which may provide it with greater tolerance compared to other cereal crops [22].

### 2.4. Water Use Efficiency

Water use efficiency (WUE) is critical for a plant’s resilience to both salt and drought stress, as it maximizes biomass production while reducing water loss. This adaptation allows plants to thrive under limited water availability. Higher water use efficiency enables plants to generate more biomass per unit of water consumed [71]. Compared to other related grass families, such as wheat, maize, and sorghum, foxtail millet exhibits a higher water use efficiency under salt and drought stress conditions [72,73]. In foxtail millet, water use efficiency is strongly associated with various morphological traits, including a dense root system, thickened cell walls, specific arrangements of epidermal cells, and a smaller leaf area [74]. Yang et al. [75] reported that the overexpression of the plasma membrane intrinsic protein (PIP) gene from foxtail millet (Si PIP) enhanced root water transport capacity and improved root exudation. Consequently, foxtail millet varieties with high SiPIP gene expression exhibited great drought and salt tolerance compared to those with low SiPIP gene expression.

## 3. Salinity Stress Response

**Figure 2:**
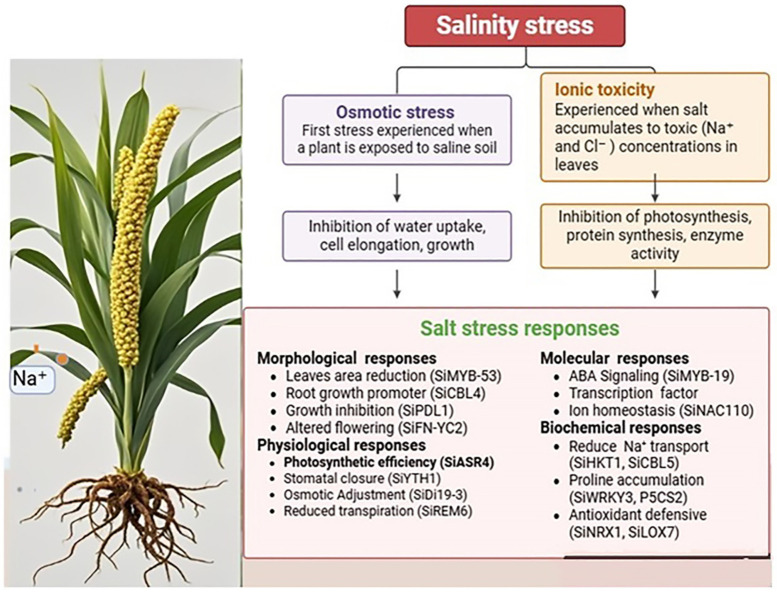
Salt stress response mechanisms in foxtail millet: Foxtail millet salt tolerance is a trait regulated by the coordinated expression of various genes (indicated in the brackets). These genes help the plant to manage ion balance, osmotic stress, oxidative stress, and cellular damage, thereby ensuring survival and productivity under saline conditions.

Soil salinity presents a significant challenge to many crops; however, foxtail millet demonstrates notable tolerance to saline environments due to its ability to regulate ion balance and mitigate oxidative stress [40]. Evaluating foxtail millet’s response to salt stress involves assessing different morphological, physiological, biochemical, and molecular parameters. These parameters elucidate the mechanisms through which the plant endures salt-induced stress and its specific adaptive responses that enable survival and growth (Figure 2). Salt stress triggers the production of reactive oxygen species (ROS) [76], and their accumulation can disrupt macromolecules and cellular signaling pathways, leading to cellular autophagy [77]. Various methodologies are employed to investigate the physiological and molecular mechanisms underlying salt tolerance in foxtail millet, with the goal of enhancing agricultural productivity and sustainability [78]. A common adaptive response to salt stress is the accumulation of osmolytes, including proline, sugars, and proteins, which scavenge reactive oxygen species, thereby protecting cellular structures and maintaining membrane integrity.

In foxtail millet, the combined application of putrescine and spermidine has been shown to enhance salt tolerance by reducing hydrogen peroxide (H_2_O_2_) levels and minimizing electrolyte leakage [79]. Sudhakar et al. [80] reported that NaCl-treated foxtail millet seedlings exhibited a significant increase in proline synthesis. Proline, an osmoprotectant, accumulates substantially in foxtail millet after nine days of osmotic stress treatment [60]. The salt-tolerant foxtail millet Yugu2 variety demonstrated increased levels of organic acids, phenolic compounds, carbohydrates, and amino acids under saline stress, which are associated with the functionality of ion channels and the antioxidant defense system [81]. This suggests that the effectiveness of the antioxidant defense mechanism is directly related to the concentrations of these compounds [82].

### 3.1. Regulation of Gene Expression Under Salt Stress

Under salt stress, foxtail millet activates complex regulatory networks that modulate gene expression, facilitating adaptation to high salinity [83]. These networks control the expression of salt stress-responsive genes through mechanisms that activate or suppress specific genes, thereby maintaining ion homeostasis, osmotic balance, stress protection, and antioxidant defense. Among the transcription factors, Di19 has been identified as interacting with PLATZ to regulate salt tolerance in transgenic foxtail millet and *Arabidopsis* seedlings. The overexpression of Di19-3 enhances salt tolerance in both species by increasing the transcript levels of key genes, including Na^+^/H^+^ antiporter (*NHX*), salt overly sensitive (*SOS*), and calcineurin B-like proteins [84]. Notably, the expression of the MYB-like transcription factor gene *SiMYB19* (*Seita.1G250600.1*) in foxtail millet is significantly upregulated in response to salt stress. Its overexpression increases abscisic acid accumulation in transgenic rice by upregulating the ABA biosynthesis gene *OsNCED3* (Table 1) [85].

In foxtail millet, members of the WRKY, SPL, and TCP gene families are expressed under salt stress conditions [86,87,88]. For instance, *SiWRKY064*, *SiWRKY066*, *SiWRKY074*, and *SiWRKY082* are significantly upregulated during salinity stress, highlighting their potential roles in salt stress signaling [86]. Electrolyte leakage assays conducted on transgenic plants revealed significantly lower ion leakage under high salinity stress, indicating a notable difference between the two groups [42]. In contrast, the expression levels of *SiTCP2*, *SiTCP3*, *SiTCP4*, *SiTCP5*, and *SiTCP12* were downregulated in response to salt stress [87]. During salt stress, *SiLOX10* expression initially decreased before increasing, whereas *SiLOX8* exhibited the opposite trend, with an initial rise followed by a decline [89]. The genes *SiCYP19-a* and *SiCYP19-b* regulate salt stress tolerance in foxtail millet [90]. Notably, *SiCYP19-b* is associated with a significant increase in proline content and reduced reactive oxygen species (ROS) accumulation compared to *SiCYP19-a*.

**Table 1:** Protein and transcription factors involved in foxtail millet salt stress response.

Name of Protein/TF	Gene Name	Functions	Reference
Abscisic acid stress and ripening induction	*SiASR4*	Reduce ROS accumulation. Increase chlorophyll content.	[43]
Later embryogenesis abundant	*SiLEA*	Increases proline and soluble sugar.	[62]
Thioredoxin (TRX)	*SiNRX1*	Promotes antioxidant enzyme (CAT, SOD, and POD) activity system.	[63]
Alternative splicing	*SiCYP19-b*	Increases the proline content and reduces ROS accumulation.	[90]
Nuclear Factor Y	*SiNF-YA1*	Increases ROS scavenging and enhances the antioxidant system.	[44]
	*SiNF-YB8*	Enhances antioxidant system and increases ROS scavenging.	[44]
	*SiFN-YC2*	Regulates early flowering.	[46]
PLATZ	*SiPLATZ12*	Negatively regulates salt tolerance by lowering Na^+^/H^+^ antiporter activities of SOS1 and NHXs.	[91]
Cys2/His2-type	*SiDi19-3*	Upregulates NHX, SOS, and CBL genes under salt stress.	[84]
High-affinity K^+^ transporter (HKT) transporter	*SiHKT1*	Minimizes Na^+^ transfer from roots to shoots and Na^+^ accumulation in shoots.	[81]
DREB	*SiARDP*	Promotes proline accumulation	[42]
PTI1	*SiPTI1-5*	Regulates dynamic ROS balance.	[92]
REM	*SiREM6*	Reduces electrolyte leakage and increases proline accumulation.	[93]
LOXs	*SiLOX7*	Promote antioxidant enzyme activities.	[89]
PLETHORA-LIKE 1	*SiPLT-L1*	Promotes primary root growth.	[94]
MYB	*SiMYB19*	Regulates ABA synthesis and signal transduction.	[85]
	*SiMYB16*	Increases the flavonoid and lignin contents and activities of fatty acid synthesis enzymes. Regulates lignin and suberin biosynthesis.	[95]
Nonspecific lipid transfer protein	*SiLTP*	Involved in the ABA-dependent transduction signal pathways. Promotes proline and soluble sugar content.	[96]
Δ1-pyrroline-5-carboxylate synthetase	*SiP5CS2*	ROS scavenging.	[60]
Calcineurin B-like protein	*SiCBL5*	Lower Na^+^ accumulation and stronger Na^+^ efflux.	[97]
Calcineurin B-like protein	*SiCBL4*	Enhances root growth.	[98]
CIPK	*SiCIPK24*	Promotes root elongation.	[98]
C-terminal-encoding peptides	*SiCEP3*	Promote ABA import.	[99]
bZIP	*SibZIP67*	Reduces malondialdehyde, enhances antioxidant enzyme activities.	[100]
WRKY	*SiWRKY3*	Reduces oxidative stress and proline contents.	[101]
NAC	*SiNAC110*	Regulates proline biosynthesis, ion homeostasis, and ABA signaling pathway-independent osmotic balance	[40]

### 3.2. Ionic Balance Maintenance

Maintaining ionic balance under salt stress is essential for the survival of foxtail millet and other plants in saline environments. High salinity poses two primary challenges for plants: osmotic stress, caused by an excess of salts outside the roots, and ionic stress, resulting from the accumulation of harmful ions such as sodium (Na^+^) and chloride (Cl^−^) within the plant. Foxtail millet manages ionic balance under salt stress through mechanisms including Na^+^ exclusion, K^+^ retention, ion compartmentalization, osmotic adjustment, and antioxidant defenses, all regulated by complex hormonal signaling and changes in gene expression [102]. These strategies enable foxtail millet to adapt to saline environments and mitigate ion toxicity. Salinity disrupts plant growth and development by causing excessive uptake of Na^+^ and Cl^−^, leading to nutritional imbalances [81,103]. Foxtail millet maintains lower cytoplasmic Na^+^ levels by extruding Na^+^ from cells, particularly root cells, via plasma membrane Na^+^/H^+^ antiporters such as SOS1 (salt overly sensitive 1), which exchange Na^+^ for H^+^ ions [78]. Excess Na^+^ that enters the plant is compartmentalized into vacuoles, isolating it from sensitive cellular machinery. This process is mediated by vacuole Na^+^/H^+^ antiporters, such as NHX1. Maintaining a high potassium (K^+^)/Na^+^ ratio is critical for plant survival under salt stress [104]. Potassium, essential for numerous cellular functions, is preferentially absorbed over Na^+^ by high-affinity K^+^ transporters (HKTs) [102]. This ensures sufficient K^+^ levels to sustain metabolic activities. The regulation of ion channels and transporters, which selectively manage the uptake of essential ions like K^+^ and calcium (Ca^2+^) while excluding harmful concentrations of Na^+^ and Cl^−^, is vital for maintaining plant health [102,105]. The preservation of ionic equilibrium across cellular membranes underpins the plant’s ability to tolerate salt stress [106,107].

Several salt-responsive genes and proteins are essential for ionic homeostasis maintenance (Table 1). The salt overly sensitive (SOS) pathway, which includes SOS1, SOS2, and SOS3, is a well-established signaling mechanism that regulates Na^+^ transport, enabling plants to cope with salt stress by maintaining ionic balance. Calcineurin B-like proteins (CBLs), including SOS3, play a crucial role in regulating salt uptake in plants [108]. Notably, SOS3 downregulation increases salt hypersensitivity, whereas the overexpression of SiCBL5 enhances salt tolerance in foxtail millet. Lines overexpressing SiCBL5 exhibit a lower Na^+^/K^+^ ratio compared to RNAi-SiCBL5 mutants [97]. SOS2 (CIPK24) and SOS3 (CBL4) encode a protein kinase (SOS2) and a calcium-binding protein (SOS3), respectively, which form a complex that activates SOS1. SOS3 detects calcium (Ca^2+^) signals induced by salt stress and interacts with SOS2 to phosphorylate and activate SOS1. The expression of CIPK24 and CBL4 is upregulated under salt stress, and the overexpression of SiCIPK24 in Arabidopsis improves salt tolerance [98]. Similarly, the overexpression of AtCBL5 and AtCBL1 increases salt tolerance in Arabidopsis, while their functional absence leads to heightened salt hypersensitivity in *Arabidopsis* seedlings [109,110].

### 3.3. Oxidative Responses

Salt stress triggers the production of reactive oxygen species (ROS) [76]. The oxidative response of foxtail millet under salt stress involves a complex interaction with ROS production, the activation of antioxidant defense mechanisms, and signaling pathways aimed at protecting cellular integrity from damage [111,112]. To counteract ROS accumulation, foxtail millet activates its antioxidant defense system, which includes both enzymatic and non-enzymatic components. Key antioxidant enzymes, such as class I and class III peroxidases (PODs), catalase (CAT), and superoxide dismutase (SOD), play crucial roles in scavenging ROS, including superoxide (O_2_^−^) and hydrogen peroxide (H_2_O_2_). Notably, POD activity is significantly higher in salt-tolerant varieties like Yugu1 and Qinhuang2 compared to the salt-sensitive variety AN04 [89]. Additionally, ascorbate peroxidase (APX) also plays a critical role in detoxifying H_2_O_2_ and maintaining cellular biochemical equilibrium under salt stress [113]. Furthermore, the oxidation of polyamines regulates antioxidative enzymes, which are essential for removing toxic levels of O_2_^−^ and H_2_O_2_ in foxtail millet [80]. Salt stress also activates genes involved in detoxifying ROS, including those coding for non-enzymatic antioxidants. The assessment of foxtail millet’s salt stress tolerance highlights the importance of pathways related to phenylpropanoid and flavonoid biosynthesis, as well as the roles of lysophospholipids, ascorbic acid, glutathione, and carotenoids [81,114].

## 4. Response to Drought Stress

The drought stress response of foxtail millet involves the complex interplay of physiological, biochemical, and molecular mechanisms that facilitate the plant’s survival and growth in arid and semi-arid environments. To alleviate the adverse impacts of drought, foxtail millet reduces its net photosynthetic rate by modulating stomal closure, synthesizing osmotic regulators to preserve cellular osmotic equilibrium, and generating diverse antioxidants [60,115].

Research indicates that different cultivars of foxtail millet exhibit varying physiological and biochemical responses to drought stress, including alterations in metabolite metabolism, enhanced lignin and fatty acid metabolism, and adjustments in the ratio of soluble sugars to starch. These adaptive strategies contribute to drought resilience, distinguishing them from cultivars that are more susceptible to drought conditions [75,116,117].

### 4.1. Morphological, Physiological, and Biochemical Responses

**Figure 3:**
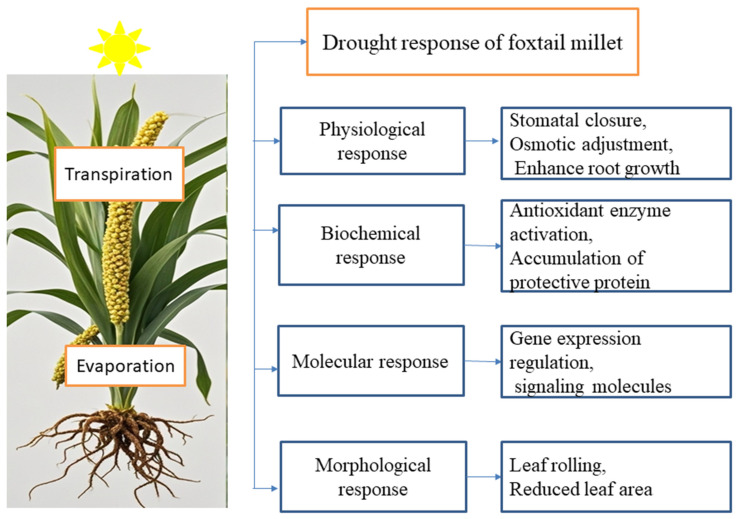
Drought stress tolerance responses in foxtail millet: In foxtail millet (a hardy and drought-tolerant crop), drought stress signaling pathways involve complex networks of morphological, molecular, biochemical, and physiological responses. These pathways enable the plant to survive and continue to grow under water-limited conditions.

Plants exhibit various morphological adaptations to drought stress, including a shorter lifespan, reduced height and leaf area, denser root system, altered flowering times, increased root length with decreased shoot length, enhanced tillering, and leaf folding (Figure 3). In foxtail millet, a key adaptation is the development of longer, denser root hairs, which form a substantial rhizosheath [29]. This adaptation increases root hair density and length. It facilitates deeper soil penetration for water uptake. While this adaptation may reduce shoot growth and photosynthetic pigment content, it is associated with the enhanced accumulation of compatible solutes, a mechanism that contributes to improving drought tolerance [118,119]. Ultimately, these changes help foxtail millet maintain better hydration and sustain growth under drought conditions.

The physiological response of foxtail millet to drought stress involves mechanisms (Figure 3) that enable the plant to cope with water scarcity while maintaining growth and survival [20]. Under conditions of drought, foxtail millet primarily engages in the synthesis of protein associated with stress response mechanisms, antioxidant defense systems, the production of osmoprotectants, the regulation of photosynthesis, hormone signaling pathways, and modifications to the cell wall [20,73]. These responses reduce water loss, optimize water use, and support metabolic functions during water scarcity. According to Shinozaki and Yamaguchin-shinozaki [120], a protein synthesized as a result of gene expression in response to drought stress can be categorized into two distinct groups. The first group comprises proteins that may provide direct protection to plant cells against damage associated with stress, such as late embryogenesis abundant proteins. The second group consists of protein factors involved in regulating signal transduction and gene expression, which contribute to the plant’s stress response, including protein kinases [22,121].

Protein levels in plant tissues decrease under water deficiency, which can be attributed to either protein breakdown or reduced synthesis [122]. However, the synthesis of specific proteins, particularly those involved in drought stress responses, such as late embryogenesis-abundant proteins (LEAPs), protein kinases, dehydrins, osmotins, and heat shock proteins, responsible for osmoprotectant biosynthesis and detoxification, may continue [123,124,125,126]. This suggests that proteins play a crucial role in regulating cellular water status, though they do not directly regulate water. Under drought stress, LEA protein expression is enhanced to prevent the denaturation and aggregation of target proteins [127]. These proteins may act as hydrating buffers, reducing water loss during drought, osmotic, or freezing stress, which is vital for maintaining the functionality of desiccated cells [128]. LEA proteins are reportedly crucial for stabilizing membrane proteins and assisting in osmotic adjustment [129]. In the drought-tolerant Jigu39 cultivar of foxtail millet, the leaf-soluble protein content increased significantly following drought stress, whereas the drought-susceptible Longgu16 cultivar showed no notable change in soluble protein levels [130].

Protein kinases play a crucial role in drought tolerance in plants, including foxtail millet, by regulating cellular responses to water scarcity [115]. These enzymes participate in signal transduction pathways that help the plant to sense and respond to drought stress. When exposed to drought conditions, protein kinases activate specific stress-responsive genes and proteins through phosphorylation (the addition of a phosphate group) [131,132]. This process triggers pathways that help the plant adapt to water deficiency. DROOPY LEAF1 (DPY1) is a protein kinase essential for the acclimation of foxtail millet to drought stress. During osmotic stress, DPY1 undergoes phosphorylation and activation, contributing to over 50% of the global phosphorylation events triggered by osmotic stress. This includes the phosphorylation of SnRK2 kinases, which are key players in the osmotic stress response. DPY1 functions as an upstream regulator of Stress-Activated Protein Kinase 6 (SAPK6), a part of the subclass I SnRK2. The full activation of SAPK6 is necessary for regulating downstream genes that initiate the drought stress response [133].

Foxtail millet exhibits various adaptations to regulate the balance between soluble sugars and starch under drought stress [134]. Drought-tolerant foxtail millet varieties strategically allocate photosynthetic sugars to the roots during drought conditions [28] through enhanced sugar transport mechanisms and increased beta-amylase activity. These adaptations are critical for controlling the soluble sugar-to-starch ratio, preventing the excessive accumulation of soluble sugars [135]. Such regulation is vital for maintaining cellular hydration and stability during drought stress [75,117]. Additionally, drought-tolerant foxtail millet enhances lignin metabolism and promotes fatty acid biosynthesis, which can be converted into cutin and wax [75,117]. This metabolic pathway may play a key role in preserving osmotic stability and reducing water loss in plant cells.

### 4.2. Regulation of Gene Expression Under Drought Stress

Transcriptomic analyses of foxtail millet have identified numerous drought-inducible genes with diverse functions (Table 2), providing valuable insights into the mechanisms and regulatory networks governing drought responses in this species [60,136]. These analyses of drought-resistant cultivars have underscored the significance of enzymes such as glutamine synthetase and pyrroline-5-carboxylase in enhancing drought tolerance [130,137,138]. For example, the genes encoding ∆1-pyrroline-5-carboxylate synthetase, SiP5CS1 and SiP5CS2, are significantly upregulated in foxtail millet under drought stress [60]. Regulating these genes is crucial for preserving water balance, reducing oxidative damage, and improving drought resilience in foxtail millet. Similarly, under drought stress, the expression of the 9-cis-epoxy carotenoid dioxygenase (NCED) gene is significantly upregulated in foxtail millet. Moreover, the ectopic overexpression of SiNCED1 may enhance drought resistance by increasing endogenous abscisic acid (ABA) levels and promoting stomatal closure [139]. In comparison to rice and *Arabidopsis*, foxtail millets exhibit a great expression of genes in response to drought stress. This enhanced gene expression may confer a superior ability for foxtail millet to withstand water-deficit conditions relative to both rice and *Arabidopsis* [23,140].

However, most drought-resistant genes identified to date have been derived from short-term drought stress simulations, which may not fully capture the mechanisms involved in the plant’s drought response. Therefore, exposing foxtail millet to prolonged soil water-deficit conditions and conducting transcriptome sequencing could yield deeper insights into the regulatory networks controlling drought resistance in this crop [5,138,141].

**Table 2:** Transcription factors and proteins involved in foxtail millet drought tolerance.

Name of Protein/TF	Gene Name	Functions	Reference
Abscisic acid stress repining	*SiASR1*	Reduces ROS accumulation. Improves antioxidant enzyme activities.	[142]
miRNA	*SimiR396d*	Regulates root growth.	[143]
YTH	*SiYTH1*	Reduces excessive H_2_O_2_ accumulation. Reduces stomatal closure.	[144]
Phospholipase D	*SiPDL* *α1*	Reduces ion leakage, chlorosis, and growth inhibition.	[145]
MYB	*SiMYB56*	Regulates ABA signaling pathways and lignin biosynthesis.	[146]
	*SiMYB53*	Regulates plant growth and development. Provides drought stress signals.	[46]
Lateral organ boundaries domain (LBD)	*SiLBD21*	Involved in root development.	[147]
Δ1-pyrroline-5-carboxylate synthetase	*SiP5CS2*	Promotes proline accumulation.	[60]
Autophagy-related gene	*SiATG8a*	Increases chlorophyll and proline contents. Reduces malondialdehyde content.	[148]
NAC	*SiNAC110*	Regulates proline biosynthesis, ion homeostasis, and ABA signaling pathway-independent osmotic balance.	[40]
9-cis-epoxycarotenoid dioxygenase (NCED)	*SiNCED1*	Modulates ABA biosynthesis and enhances ABA accumulation	[139]
Calcium-dependent protein kinases (CDPKs)	*SiCDPK24*	Activate drought stress response gene expression and promote drought stress recovery	[132]

### 4.3. Oxidative Response to Drought Stress

Under conditions of water stress, plants generate reactive oxygen species (ROS), which can damage biomolecules such as carbohydrates, proteins, lipids, and nucleic acids. This damage leads to a reduction in photosynthesis, respiration, and overall plant growth [149,150]. Foxtail millet demonstrates a robust response to drought stress by enhancing both enzymatic and non-enzymatic antioxidant systems, which are vital for scavenging reactive oxygen species and reducing oxidative damage. This adaptive mechanism is essential for maintaining cellular integrity and function under stress conditions [117,151]. Avashthi et al. [151] found that the expression level of reactive oxygen species (ROS) scavenging genes in foxtail millet under drought stress is higher than those in rice and sorghum. This indicates that, under drought stress, foxtail millet activates an antioxidant enzyme system to counteract the accumulation of free O_2_ radicals and H_2_O_2_, thereby preserving normal growth and development. These enzymes collaborate to alleviate oxidative damage caused by drought [60,152,153].

Under drought stress, the plant antioxidative system includes ROS scavenging enzymes, with superoxide dismutase (SOD), peroxidase (POD), and catalase (CAT) playing key roles in eliminating ROS and working together to mitigate oxidative damage caused by the stress [153]. Notably, Qin et al. [60] identified eight genes related to the ROS system that were upregulated in their studies, along with increased activities of SOD, POD, and CAT in foxtail millet, all of which contributed to drought tolerance [60]. Research highlights the crucial role of SOD genes in plant adaptation to abiotic stress. In foxtail millet, the expression of all SiSOD genes decreased after 1 h of drought stress, followed by an increase. Subsequently, the expression levels of SiFSD1, SiFSD2, and SiMSD were significantly elevated under drought conditions [70]. Based on their metal cofactors, SOD proteins are classified into three types: manganese SOD (MnSOD), copper–zinc SOD (Cu/Zn-SOD), and iron SOD (FeSOD) [154,155].

### 4.4. Relative Water Content

Relative water content (RWC) is an important indicator of a plant’s water status and helps assess the degree of drought stress. Drought stress can be classified into three levels based on the relative water content: mild stress (water loss of 8–10%), moderate stress (water loss of 10–20%), and severe stress (water loss of more than 20%) [156]. These categories are useful for determining how different genotypes respond to drought conditions. Boominathan et al. [157] identified drought-tolerant foxtail millet genotypes by evaluating their relative water content under drought stress. In this study, the genotypes Ise27, PS4, AP4, Ise138, and Ise174 exhibited lower water loss and high relative water content compared to drought-susceptible genotypes. Similarly, Suneetha et al. [158] used relative water content as a criterion to screen drought-tolerant foxtail millet genotypes. Foxtail millet genotypes with low relative water content undergo a loss of turgidity, causing stomatal closure and reduced photosynthesis rates. In contrast, genotypes with high relative water content maintain better stomatal conductance and photosynthesis rates, which ultimately contributes to improved yield [159,160]. Under drought stress, transgenic rice plants expressing the SiWLIM2b gene from foxtail millet demonstrated higher relative water content compared to the wild type of rice. This suggests that the overexpression of the foxtail millet SiWLIM2b gene enhances drought tolerance in transgenic rice [161]. Other scholars have indicated that the expression of the SiSWEET6 and SiA-INV genes plays a role in regulating the relative water content in foxtail millet, particularly during drought stress conditions. These genes contribute to the maintenance of the relative water content indirectly by helping the plant sustain osmotic balance and sugar homeostasis, which are crucial for maintaining hydration and turgor pressure in the plant [134]

## 5. Conclusions and Future Prospective

Salt stress and drought stress are major consequences of climate change that pose considerable challenges to global agriculture. The lack of crop varieties that are tolerant to salinity and drought, coupled with a limited understanding of the underlying mechanisms, hampers the rehabilitation of salt-affected land, thereby threatening agricultural productivity [162,163,164]. Researchers are actively investigating plant mechanisms of salt and drought tolerance to address these challenges. Foxtail millet, renowned for its high resilience and drought tolerance, is particularly valuable in semi-arid and arid regions. This species employs a range of physiological, biochemical, and molecular strategies to withstand salt and drought stress. While both stresses induce water deficits in plants, foxtail millet exhibits distinct responses to each. Some physiological and biochemical responses may overlap, but the mechanisms for coping with salinity and drought stress differ due to the unique challenges posed by each. For example, drought stress triggers osmotic adjustment through the accumulation of osmolytes such as proline and sugars, while salt stress necessitates osmolyte production and ion compartmentalization to balance Na^+^ and K^+^ ions. Overall, foxtail millet’s ability to endure salt and drought stress results from a combination of molecular mechanisms, including gene regulation, ion homeostasis, osmotic adjustment, antioxidant defense, and hormonal signaling pathways.

**Figure 4:**
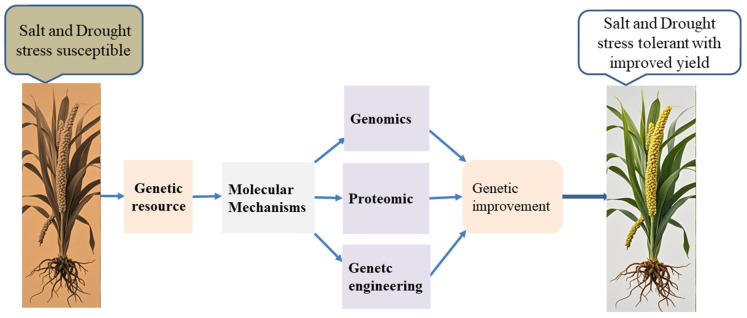
Proposed direction for developing high-yield foxtail millet varieties with excellent salt and drought tolerance.

Although numerous studies have examined the abiotic stress responses of foxtail millet, the genetic mechanisms underlying its tolerance to salt and drought remain insufficiently understood. Research on the genomic, proteomic, and metabolic responses of foxtail millet to these stress conditions is less comprehensive compared to that of crops such as rice and wheat. However, the expression levels of reactive oxygen species scavenging genes in foxtail millet under salt and drought stress are higher than those in other grass families, like rice and sorghum. Therefore, identifying the pathways and genes associated with salt and drought tolerance is crucial for understanding how this crop adapts to environmental stressors. These insights will be vital for developing foxtail millet cultivars with improved tolerance, potentially through genetic engineering, to boost agricultural yield (Figure 4).
